# Application of Chaos Mutation Adaptive Sparrow Search Algorithm in Edge Data Compression

**DOI:** 10.3390/s22145425

**Published:** 2022-07-20

**Authors:** Shaoming Qiu, Ao Li

**Affiliations:** Communication and Network Laboratory, Dalian University, Dalian 116622, China; liao@s.dlu.edu.cn

**Keywords:** computer application technology, edge computing, chaotic adaptive sparrow search algorithm, convolutional auto-encoder network, data compression, hyperparameter optimization

## Abstract

In view of the large amount of data collected by an edge server, when compression technology is used for data compression, data classification accuracy is reduced and data loss is large. This paper proposes a data compression algorithm based on the chaotic mutation adaptive sparrow search algorithm (CMASSA). Constructing a new fitness function, CMASSA optimizes the hyperparameters of the Convolutional Auto-Encoder Network (CAEN) on the cloud service center, aiming to obtain the optimal CAEN model. The model is sent to the edge server to compress the data at the lower level of edge computing. The effectiveness of CMASSA performance is tested on ten high-dimensional benchmark functions, and the results show that CMASSA outperforms other comparison algorithms. Subsequently, experiments are compared with other literature on the Multi-class Weather Dataset (MWD). Experiments show that under the premise of ensuring a certain compression ratio, the proposed algorithm not only has better accuracy in classification tasks than other algorithms but also maintains a high degree of data reconstruction.

## 1. Introduction

As devices become more and more interconnected, sensor nodes will collect massive amounts of data, which not only have a lot of redundancy but also occupy massive communication bandwidth resources during the transmission process. Compressing data in advance on the edge server close to the data source can not only improve the efficiency of data transmission but also enable more flexible data processing in the cloud service center [1].

Deep learning has achieved good results in the field of data compression. Especially in the case of large amounts of data, it is better than traditional machine learning methods. Previous literature has described the shortcomings of machine learning in the field of neural network data compression [2]. In one such example, recurrent neural networks were used for data compression [3]. Although the compression rate was improved and the compression time was reduced, the quality of data recovery was reduced. Jalilian et al. [4] thoroughly studied the compression performance of deep convolutional neural networks (CNN) in iris image compression for the first time and proved that this technology is superior to all other related compression technologies. The Convolutional Auto-Encoder Network (CAEN) is a kind of deep neural network that learns without supervision and encodes data effectively. Liu et al. [5] proposed a simple and efficient CAEN structure for data compression in wireless sensor networks (WSN). In Lee et al. [6], a CAEN compression model based on frequency selection was proposed, which improved reconstruction quality while maintaining the compression ratio (CR). In an edge server, CAEN is used to encode and compress the data and send it to the cloud service center, which can be directly used for machine learning tasks, or the convolution decoder of CAEN is used to reconstruct the data in the cloud service center [7].

The hyperparameters of the network have a great impact on the quality of the final model. The setting of traditional hyperparameters usually requires a lot of manpower to tune, and it is difficult to find the optimal solution. In recent years, many researchers have been looking for suitable methods to construct optimal network hyperparameters to improve model performance. Ezzat et al. [8], using a gravitational search algorithm to determine the optimal hyperparameters of the DenseNet121 architecture, achieved high accuracy in diagnosing COVID-19 from chest X-ray images. Roselyn et al. [9] used particle swarm optimization (PSO) to optimize feature selection to improve the classification performance of the classifier. Liu et al. [10] proposed a learning algorithm based on an evolutionary membrane algorithm to optimize the neural structure and network parameters of a liquid state machine (LSM). Guo et al. [11] proposed a distributed particle swarm optimization (DPSO) method for optimizing hyperparameters to find high-performance convolutional neural networks but only compared this with a particle swarm algorithm, not with other algorithms. Tuba et al. [12] used the bare-bones fireworks algorithm to optimize the hyperparameters of the convolutional neural network and achieved good classification results in multiple network structures, proving the feasibility of the evolutionary algorithm to optimize a deep neural network, but the characteristics of the datasets used are obvious, and the practical application is not fully considered.

The Sparrow Search Algorithm (SSA) [13] is a swarm intelligence optimization algorithm proposed in recent years which has been widely used in many fields. Compared with traditional algorithms, the sparrow algorithm is simple in principle, easy to implement, and has strong optimization ability. However, the sparrow algorithm still has some limitations in aspects such as population initialization and location update strategy, resulting in the inconsistency of global search ability and local optimization ability and a weak ability to jump out of local optima. Wu et al. [14] proposed an improved Sparrow Search Algorithm (ISSA) to optimize some parameters in a Fast Random Configuration Network (FSCN) to make it have better classification performance. Liu et al. [15] proposed an improved SSA called CASSA to solve the UAV route planning problem. Nguyen et al. [16] proposed a new and improved enhanced SSA (ESSA) for optimal operation planning of power microgrids. Although these improved methods have improved the performance of the SSA to a certain extent, further research is needed to enhance the global and local search capabilities of the algorithm, improve the algorithm convergence speed, and help the algorithm escape from local optima.

Based on the above research, this paper proposes a chaotic mutation adaptive sparrow search algorithm (CMASSA), which uses the improved Circle Map to initialize the individual sparrow population to enhance the diversity of the population. Sine and cosine operators are introduced into the alerters equation to improve searchability. In the sparrow search algorithm, the ratio of discoverers and followers is generally fixed, which leads to the inconsistency between global search ability and local optimization ability. An adaptive population adjustment strategy is proposed to balance the global search ability and local optimization ability of CMASSA; In the field of compression, a new fitness function is proposed by combining the CMASSA with CAEN, and the hyperparameters are encoded with real numbers. Finally, the optimal CAEN structure is obtained under the premise of ensuring a certain data compression ratio.

The contributions of this paper are:

1. A chaotic mutation adaptive sparrow search algorithm (CMASSA) is proposed.
2. A new fitness function is proposed to apply CMASSA to CAEN data compression under edge computing architecture.
3. The simulation results show that the CMASSA outperforms other algorithms of the same type when optimizing 10 benchmark functions. In the data compression problem, the performance of CMASSA on MWD far outperforms other optimization algorithms.

The rest of the paper is structured as follows. Section 2 summarizes related techniques. Section 3 describes the relevant details of CMASSA. Section 4 presents the specific details of CMASSA optimizing CAEN in the context of edge computing. Section 5 discusses the experimental results. Section 6 concludes this paper.

## 2. Related Work

### 2.1. Convolutional Auto-Encoder Network

The idea of the Auto-Encoder Network (AEN) comes from sparse coding. It trains the network model through backpropagation to make the output of the model equal to the input. It is a data compression algorithm. This paper combines the idea of the AEN with a CNN and uses a convolution layer instead of a full connection layer to retain the image feature information better and build the CAEN. The network consists of two parts: a Convolutional Coding Network (CCN) and a Convolutional Decoding Network (CDN).

**Figure 1:**
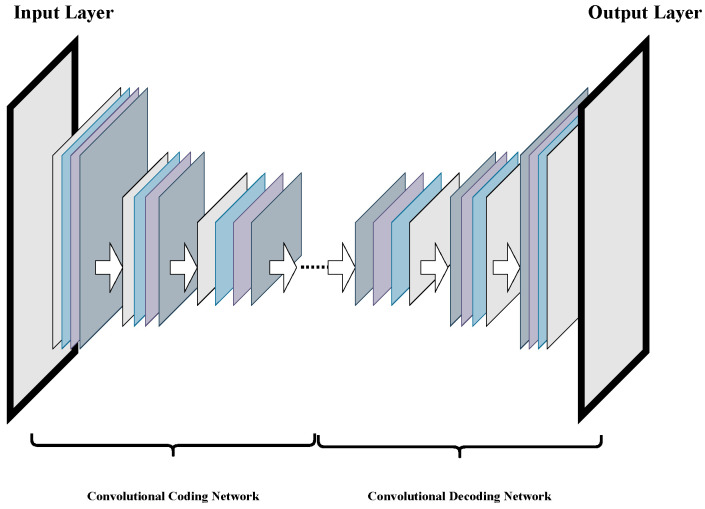
Schematic diagram of CAEN structure.

Figure 1 shows the network structure of CAEN.

CCN uses the convolution operation of the convolution layer to extract features from data and uses a pooling layer to reduce the number of parameters in the network. CDN is the inverse process of CCN, which consists of a deconvolution layer and an upper sampling layer. The upper sampling layer is opposite the pooling layer in the convolution encoder. It can make the size of the output data of each layer larger than the size of the input data, restore the feature map extracted by the encoder to the same size as the original data, and realize data reconstruction. The deconvolution layer controls the recovery of data features corresponding to the convolution layer in the convolution encoder.

### 2.2. Sparrow Search Algorithm

The SSA simulates the three foraging behaviors of sparrows and performs global search and local optimization in the search space to ensure the accuracy of the algorithm. The sparrows are divided into discoverers, followers, and alerters. The discoverer is responsible for searching the places with dense food in the space to provide direction for the whole population. Other sparrows, as followers, gain food by following the discoverer. In addition, 10–20% of the sparrows in the population are selected as the discoverer or follower, and at the same time, they act as alerters responsible for giving early warning in case of danger, updating their location, and flying to a better location.

Suppose there are *n* sparrow individuals in a sparrow population, and these sparrows search for food in a *d*-dimensional space.

(1) $$X=\left[\begin{array}{cccc} x_{1,1} & x_{1,2} & \ldots & x_{1,d}\\ x_{2,1} & x_{2,2} & \ldots & x_{2,d}\\ \vdots & \vdots & \vdots & \vdots \\ x_{n,1} & x_{n,2} & \ldots & x_{n,d} \end{array}\right]$$

where $x_{i,d}$ represents the spatial position of the *i*-th sparrow in the *d* th solution dimension.

The discoverers location is updated as follows:

(2) $$X_{i,j}^{t+1}=\left\{\begin{array}{ccc} X_{i,j}^{t}\cdot \exp\left(-\frac{i}{\alpha \cdot T_{\max}}\right) & \;\mathrm{if}\; & AT<ST\\ X_{i,j}^{t}+Q\cdot L & \;\mathrm{if}\; & AT\geq ST \end{array}\right.$$

where $X_{i,j}^{t}$ is the position of sparrow *i* in iteration *t* in dimension *j* of the search space. $T_{\max}$ indicates the maximum number of iterations of the sparrow algorithm. α is a random number within [0, 1) subject to a uniform distribution. *Q* is a random number in [0, 1) that obeys a normal distribution. *L* is a one-dimensional vector of length *d* whose elements are all ones. AT and ST represent the warning value and the safety value, respectively. AT is a random number in [0, 1). ST is used as a warning threshold. Once AT exceeds ST, sparrow individuals will update their positions to other safer places at a pace that conforms to the normal distribution.

The followers’ location is updated as follows:

(3) $$X_{i,j}^{t+1}=\left\{\begin{array}{cc} Q\cdot \exp\left(\frac{X_{worst}^{t}-X_{i,j}^{t}}{i^{2}}\right) & \;\mathrm{if}\;\;i>n/2\\ X_{p}^{t+1}+\left|X_{i,j}^{t}-X_{P}^{t+1}\right|\cdot A^{+}\cdot L & \;\mathrm{otherwise}\; \end{array}\right.$$

where $X_{worst}^{t}$ is the global worst position in the current iteration, $X_{P}^{}$ is the optimal position currently occupied by the discoverer, $A^{+}=A^{T}{\left(AA^{T}\right)}^{-1}$, and *A* is a one-dimensional vector of length *d* whose elements are all 1 or −1. When i>n/2, it means that the sparrow individual gets little food as a follower; in such a case, the sparrow individual will update its position with the law of normal distribution to get more food. Otherwise, they jump to the status of discoverer for food.

The alerters’ location is updated as follows:

(4) $$X_{i,j}^{t+1}=\left\{\begin{array}{cc} X_{best}^{t}+\beta \cdot \left|X_{i,j}^{t}-X_{best}^{t}\right| & \;\mathrm{if}\;\;f_{i}>f_{g}\\ X_{i,j}^{t}+K\cdot \left(\frac{|X_{i,j}^{t}-X_{worst}^{t}|}{\left(f_{i}-f_{w}\right)+\varepsilon }\right) & \;\mathrm{if}\;\;f_{i}=f_{g} \end{array}\right.$$

where $f_{i}$ is the fitness value of the individual sparrow *i*, $f_{g}$ is the optimal individual fitness value of sparrow in this iteration, $f_{w}$ is the worst individual fitness value of the sparrow in this iteration, $X_{best}^{t}$ is the global optimal position in this iteration, β is a set of random numbers of length *d*, mean 0, and variance 1, normally distributed within [0, 1), *K* is a random number in the interval [−1, 1], and ε is a minimum constant to prevent the current individual’s fitness from being the worst global fitness value, resulting in a denominator of 0. When $f_{i}>f_{g}$, the individual sparrow is in the outermost part of the whole sparrow population. At this time, the individual will move to the best position. When $f_{i}=f_{g}$, it indicates that the sparrow individuals in the middle of the population have realized the danger and will move to other positions.

## 3. Chaos Mutation Adaptive Sparrow Search Algorithm

### 3.1. Improved Circle Map Initialization Population

**Figure 2:**
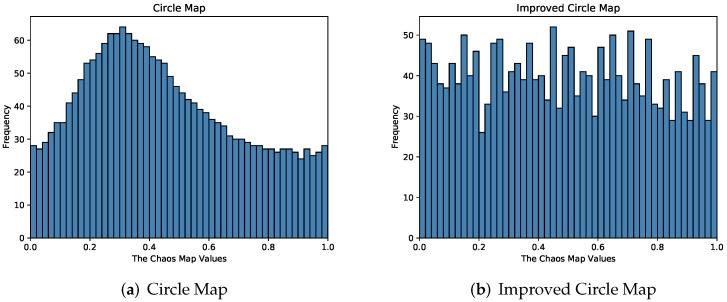
Histogram of Circle Map Frequency distribution before and after improvement.

The population initialization of SSA adopts a set of random sample values that obey a uniform distribution in [0, 1). Chaos theory is widely used in swarm intelligence algorithms due to its randomness and non-repetition. Compared with random search, chaos theory can make full use of the search space, so it is often used to enhance the diversity of the initial population and improve the algorithm to achieve optimized performance [17,18,19,20]. Circle Map maps the variables to the value interval of the chaotic variable space and finally transforms the solution linearly into the optimization variable space. It has the characteristics of unpredictability and aperiodicity, which can be used to improve the performance of the algorithm. Circle Map values are between [0, 1].This paper considers introducing the Circle Map into the population initialization of SSA, so that sparrow individuals can search the search space more thoroughly. However, as shown in Figure 2a, the Circle Map still has the problem of dense values between [0.2, 0.5] and uneven distribution, so the Circle Map is slightly improved to make the chaotic value distribution more uniform.

The mathematical model for introducing the Circle Map into the SSA population initialization is:

(5) $$\begin{array}{cc} \; & a_{i,j}^{t}=rand\left(X_{lb}^{j},X_{ub}^{j}\right)\\ \; & X_{i,j}^{t}=\left(a_{i,j}^{t}+0.2-\left(\frac{0.5}{2\pi }\sin\left(2\pi \cdot a_{i,j}^{t}\right)\right)\right)\;\mathrm{mod}\;1. \end{array}$$

where $X_{lb}^{j}$ and $X_{ub}^{j}$ represent the lower and upper bounds of the *j*-th sparrow search space, respectively. $a_{i,j}^{t}$ is the position of the *i*th sparrow in SSA in dimension *j* of the search space at iteration *t*.

The improved Circle Map expression is:

(6) $$X_{i,j}^{t}=\left(3.845a_{i,j}^{t}-\left(\frac{0.69}{3.845\pi }\sin\left(3.84\pi \cdot a_{i,j}^{t}\right)\right)\right)\;\mathrm{mod}\;1.$$

where the newly added parameters are obtained through experiments and manual tuning, and Figure 2 demonstrates the effectiveness of the results.

The Frequency distribution histogram of the Circle Map mapping function before and after improvement is shown in Figure 2.

As can be seen from the Figure 2, the improved Circle Map value Frequency distribution is more uniform. Therefore, the improved Circle Map is used to initialize the population to enhance the diversity of the population and increase the algorithm optimization ability.

### 3.2. Sine and Cosine Mutation Operator Update Position

The Sine-Cosine Algorithm (SCA) [21] is a new global optimization algorithm proposed in recent years. It is different from other swarm intelligence optimization algorithms inspired by biological mechanisms. It mainly uses the mathematical properties of sine and cosine functions to find the optimal solution. Previous studies [22,23,24,25] introduced the sine and cosine mutation operator to strengthen the population position update and achieved good results. In the later stage of SSA iteration, the sparrow population will quickly gather near the optimal solution, resulting in serious population convergence and stagnation of the algorithm, thereby increasing the probability of the algorithm falling into a local optimum. To resolve this problem, the sine-cosine algorithm is introduced into the sparrow alerters equation to improve the local search ability of the sparrow alerters; that is, the position update in Equation (4) is changed to Equation (7):

(7) $$X_{i,j}^{t+1}=\left\{\begin{array}{cc} \left(X_{best}^{t}+\beta \cdot \left|X_{i,j}^{t}-X_{best}^{t}\right|\right)\cdot \sin r_{1}+2\sin r_{1}\cdot \left|r_{2}X_{best}^{t}-X_{i,j}^{t}\right|\cdot \left(1-\frac{t}{T_{\max}}\right) & \;\mathrm{if}\;\;f_{i}>f_{g}\\ \left(X_{i,j}^{t}+K\cdot \left(\frac{|X_{i,j}^{t}-X_{worst}^{t}|}{\left(f_{i}-f_{w}\right)+\varepsilon }\right)\right)\cdot \cos r_{1}+2\cos r_{1}\cdot \left|r_{2}X_{best}^{t}-X_{i,j}^{t}\right|\cdot \left(1-\frac{t}{T_{\max}}\right) & \;\mathrm{if}\;\;f_{i}=f_{g} \end{array}\right.$$

where $r_{1}$ is a random number with uniform distribution on [0, 2π], which is used to control the search distance of the algorithm, $r_{2}$ is a random number with uniform distribution on [0, 2];

### 3.3. Adaptive Population Adjustment Strategies

In SSA, the location update of sparrows is mainly determined by the discoverers in the sparrow population, and the number of discoverers in SSA is fixed, usually set to 10–20%. In early iterations, if the number of discoverers is relatively small, the search space cannot be fully searched globally; in the later stage of iteration, if the number of discoverers is relatively large and the number of followers is relatively small, it is easy to fall into a local optimum. To this end, this paper proposes an adaptive update of the numbers of discoverers and followers. In the early stage of algorithm iteration, more sparrow individuals are allocated as discoverers for extensive global search; in the later stage of algorithm iteration, more sparrow individuals are allocated as followers for accurate local search.

**Figure 3:**
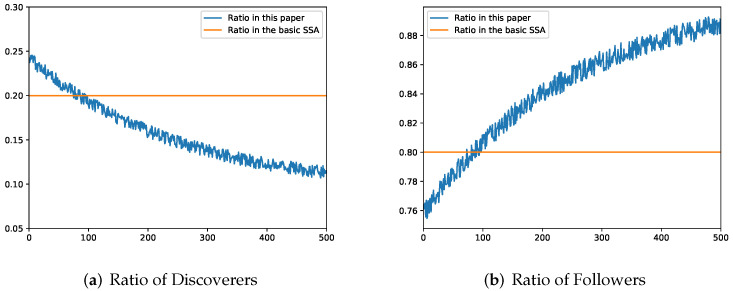
Changes in the ratio of discoverers and followers of CMASSA.

The equation for adjusting the number of discoverers and followers is:

(8) $$\begin{array}{cc} \; & r=0.15\cdot \left(2e^{-\left(2t/T_{\max}\right)}-0.1k\right)+0.1\\ \; & pNum=r\cdot N\\ \; & sNum=(1-r)\cdot N \end{array}$$

where *N* is the total population, *k* is a random number in [0, 1), used to disturb the nonlinear decreasing *r*, pNum is the number of discoverers, and sNum is the number of followers. As shown in Figure 3, the ratio of the number of discoverers and followers proposed in this paper gradually converges to 0.1 with the iteration, which can achieve a balance between the early global search and the later local optimization.

Algorithm 1 provides pseudocode for CMASSA.

**Algorithm 1:** Pseudo Code of CMASSA**Input:** Objective function f(x), sparrow population size *N*, Maximum number of iterations $T_{\max}$, Warning threshold of ST, Number of alerters vNum.
1:Initialization population using Equation (6)2:**while***t* < $T_{\max}$ **do**3:   **Calculate** pNum and sNum using Equation (8)4:   **Calculate** Random number AT5:   **for** *i* = 0: pNum **do**6:     Update position using Equation (2)7:   **end for**8:   **for** *i* = 0: sNum **do**9:     Update position using Equation (3)10:   **end for**11:   **for** *i* = 0: vNum **do**12:     Update position using Equation (7)13:   **end for**14:**end while**
**Output:** the global optimal solution

## 4. Data Compression Model

### 4.1. Cloud Edge Data Compression Architecture

**Figure 4:**
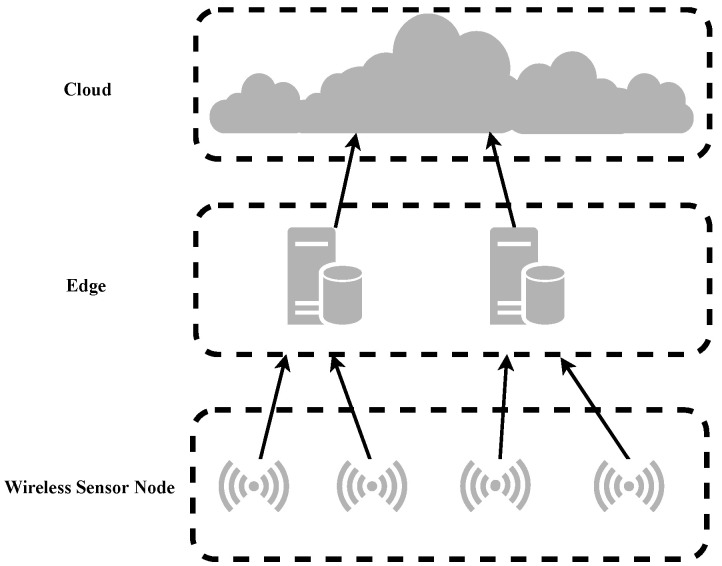
Edge computing architecture diagram.

Edge computing transfers part or all of the data processing tasks on traditional terminal devices to edge devices for execution. While ensuring low latency, it avoids uploading all data to the cloud service center and reduces bandwidth pressure. It has a better effect than the cloud computing model on tasks with high real-time response requirements, as shown in Figure 4 for the edge computing architecture diagram.

Due to the high cost of optimizing deep learning networks by swarm intelligence optimization algorithms, the processing capacity and storage space of sensor networks under edge computing are limited. Therefore, the algorithm should be trained and optimized based on historical data in the resource-rich cloud service center, and then the trained model should be delivered to the edge server close to the lower layer of edge computing. The edge server collects massive data from the underlying wireless sensor network nodes and performs data compression on the edge server according to the network model issued by the cloud service center. Sending the compressed data to the cloud service center can be directly used for machine learning tasks, or it can be used for storage or other tasks after the compressed data is restored and reconstructed in the cloud service center.

**Figure 5:**
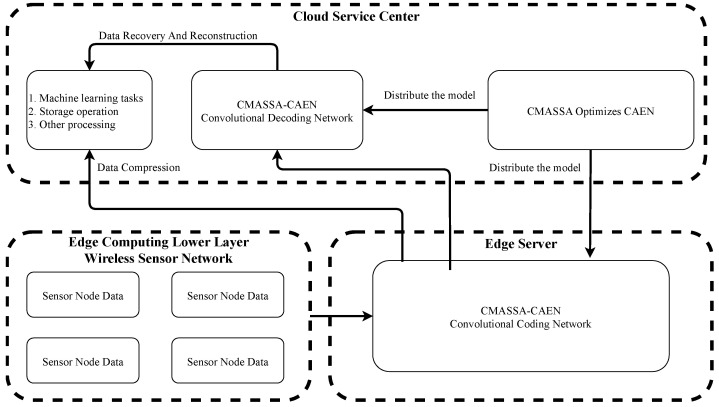
Cloud edge data compression model diagram.

The cloud-edge data compression model is shown in Figure 5.

### 4.2. CMASSA-CAEN

The data compressed by CCN will be transmitted to the cloud service center, perform other tasks, or be restored and reconstructed in the CDN. During the mathematical modeling, considering that the CMASSA selects the sparrow individual with a low fitness function value as the optimal individual of the algorithm and uses this to carry out the next update iteration, it is decided to place attention on the following two aspects when performing the modeling.

The Loss of the CCN classification task on the test set in the CAEN model is selected as one of the important indicators of the fitness function. The Loss equation is:

(9) $$Loss=1-\frac{TP+TN}{TP+TN+FP+FN}$$

where TP, FP, TN, and FN, respectively, represent the number of true positive, false positive, true negative, and false negative samples. The lower the value of Loss, the higher the classification accuracy, which proves that the data loss after compression is less.

Using the cosine similarity equation, the difference between the CDN output data and the original data is used as another important index of fitness function to measure the degree of data reconstruction. The corresponding equation is:

(10) $$Rate=1-\frac{\sum_{i=1}^{n}\left(x_{i}\cdot y_{i}\right)}{\sqrt{\sum_{i=1}^{n}x_{i}^{2}}\sqrt{\sum_{i=1}^{n}y_{i}^{2}}}$$

where $x_{i}$ and $y_{i}$ are data vectors before and after compression, respectively.

Considering the accuracy of the classification task after data compression and the accuracy of data recovery and reconstruction, the fitness function of the CMASSA is set as:

(11) Fitness=a·Loss+b·Rate

where *a*, *b* are constants, and a+b=1.

The main steps of applying CMASSA to edge computing data compression are as follows:

1. Set the number of sparrow populations, the maximum proportion coefficient between the discoverer and the follower, the maximum number of iterations, the sparrow position dimension, and other basic parameters.
2. Use the floating point encoding method to encode the CAEN hyperparameters such as the kernel size of the convolutional layer in CAEN, the kernel size of the pooling layer, the proportion of loss neurons in the dropout layer, and so on. Use each hyperparameter as a dimension of the sparrow search space, and set the upper and lower bounds of the dimension.
3. Calculate the initial fitness of each individual sparrow by using the initial position of the sparrow and record the information of the current optimal and worst individual sparrows.
4. Adjust the number of adaptive populations according to Equation (8). In the current iteration cycle, the sparrow individuals with the best fitness value are selected as discoverers, and the remaining sparrow individuals are selected as followers. The position of the sparrow individual is updated by Equations (2) and (3), and the fitness value of the discoverers and the followers is calculated and recorded by Equation (11).
5. Randomly select 20% of the current sparrow population as the guards or alerter sparrows. The position is updated by Equation (8), and the fitness value of the guards is calculated by Equation (11).
6. After one iteration cycle is completed, store the information of the optimal sparrow individual.
7. Execute the algorithm to reach the maximum number of iterations. Output and store the optimization result.

**Figure 6:**
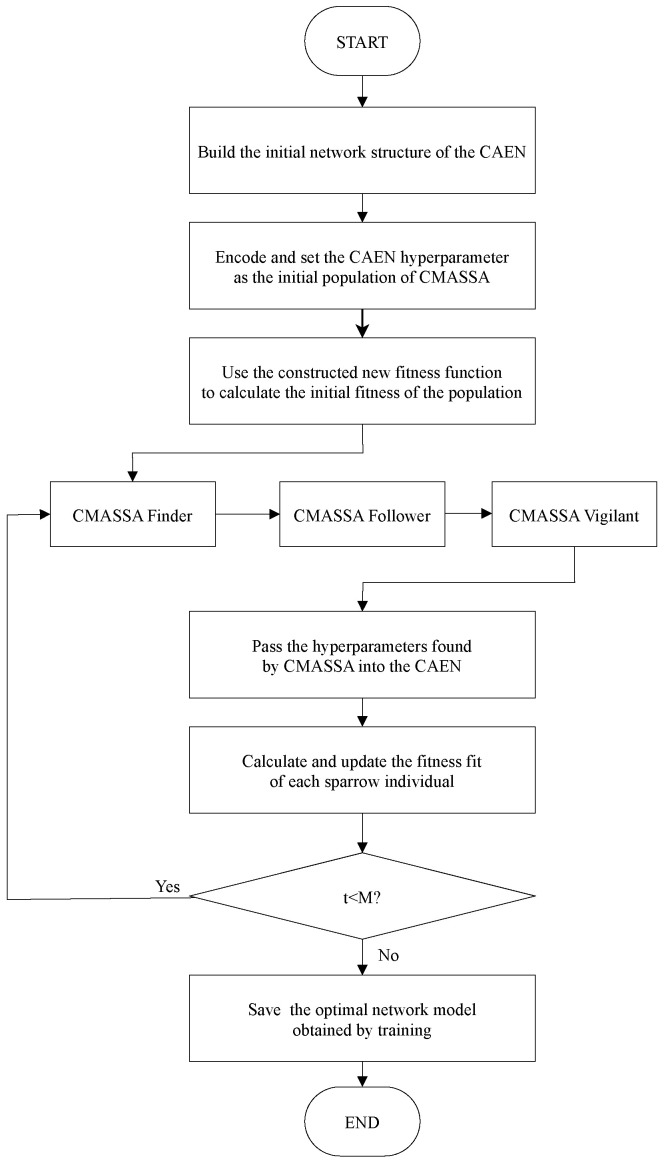
CMASSA-CAEN algorithm flow chart.

The flowchart of CAEN optimization of CMASSA is shown in Figure 6.

## 5. Simulation

### 5.1. Benchmark Function

Table 1 shows the benchmark functions used in this paper. $F_{1}$–$F_{5}$ are high-dimensional unimodal functions, and $F_{6}$–$F_{10}$ are high-dimensional multimodal functions. The high-dimensional unimodal functions have only one global optimal point and no local extreme points, mainly to test the convergence speed of the function. The multimodal function has multiple local extreme points, which are used to compare the ability of the optimization algorithm to jump out of the local optimum point and the optimization speed.

### 5.2. CMASSA Performance Comparison and Analysis

The operating environment used in the experiment is the 64-bit Windows 10 operating system, the processor type is Intel Core i5-6300H 2.3GHz, the running memory is 16 GB, and Python is used for programming.

#### 5.2.1. Effectiveness Analysis of Improvement Strategies

To prove that several strategies have a certain effect on the performance improvement of the sparrow algorithm, this paper selects the improved sparrow algorithm with a single strategy for comparison, that is: only SSA1, which improves the initialized population of the Circle Map, is introduced; only SSA2, which updates the position of the sparrow alerter by the sine and cosine mutation operator; and only SSA3, which is an adaptive population adjustment strategy. The three improved algorithms are compared with SSA and CMASSA on the benchmark function in Table 1 to verify the effectiveness of their respective strategies. The population number of the optimization algorithm is set to 30, the number of iterations is 500, the dimension and search space range are set according to the benchmark function in Table 1, and the number of alerters is set to 20% of the population, except for the CMASSA, which is set to 10%. To reduce the variability of the experiment and increase the persuasiveness of the experimental results, each function is tested 30 times, and the standard deviation (STD), the mean value (MEAN), the best value (BEST), the worst value (WORST), and the running time (TIME) are calculated from the optimal value of each run of the function.

In Table 2, STD reflects the degree of dispersion of the optimization results, MEAN reflects the stability of the algorithm, and BEST and WORST reflect the optimization ability of the algorithm. It can be seen from the data in Table 2 that in the optimization process of the benchmark function in Table 1, all improvement strategies have improved the performance of SSA to varying degrees. Among them, the improved alerter update strategy in SSA2 plays a decisive role, which not only far exceeds SSA in the calculation of the optimal value, but also has the lowest STD and MEAN except CMASSA, which proves that the algorithm has high robustness and stability. At the same time, it can be seen that other strategies also have a certain auxiliary effect on the optimization of the function. The stability of SSA1 in the benchmark function is not stronger than that of SSA, which is the result of chaotic randomness, but it is stronger than SSA in optimization ability. Although SSA3 is weaker than SSA2, it still performs better than SSA in various indicators, including time. The effectiveness of the proposed improvement strategy is proved. In the optimization process of $F_{4}$ and $F_{6}$ functions, various algorithms for improving the strategy can find the optimal value of the function.

#### 5.2.2. Compared with Other Intelligent Optimization Algorithms

To further test the optimized performance of the CMASSA, including Particle Swarm Optimization (PSO) [26], Harris Hawks Optimization (HHO) [27], Multi-Verse Optimizer (MVO) [28], Improved Sparrow Search Algorithm (ISSA) [14], Sparrow Fusion with Firefly Algorithm Search Algorithm (ESSA) [16], the optimization comparison is performed on the benchmark functions in Table 1.

**Figure 7:**
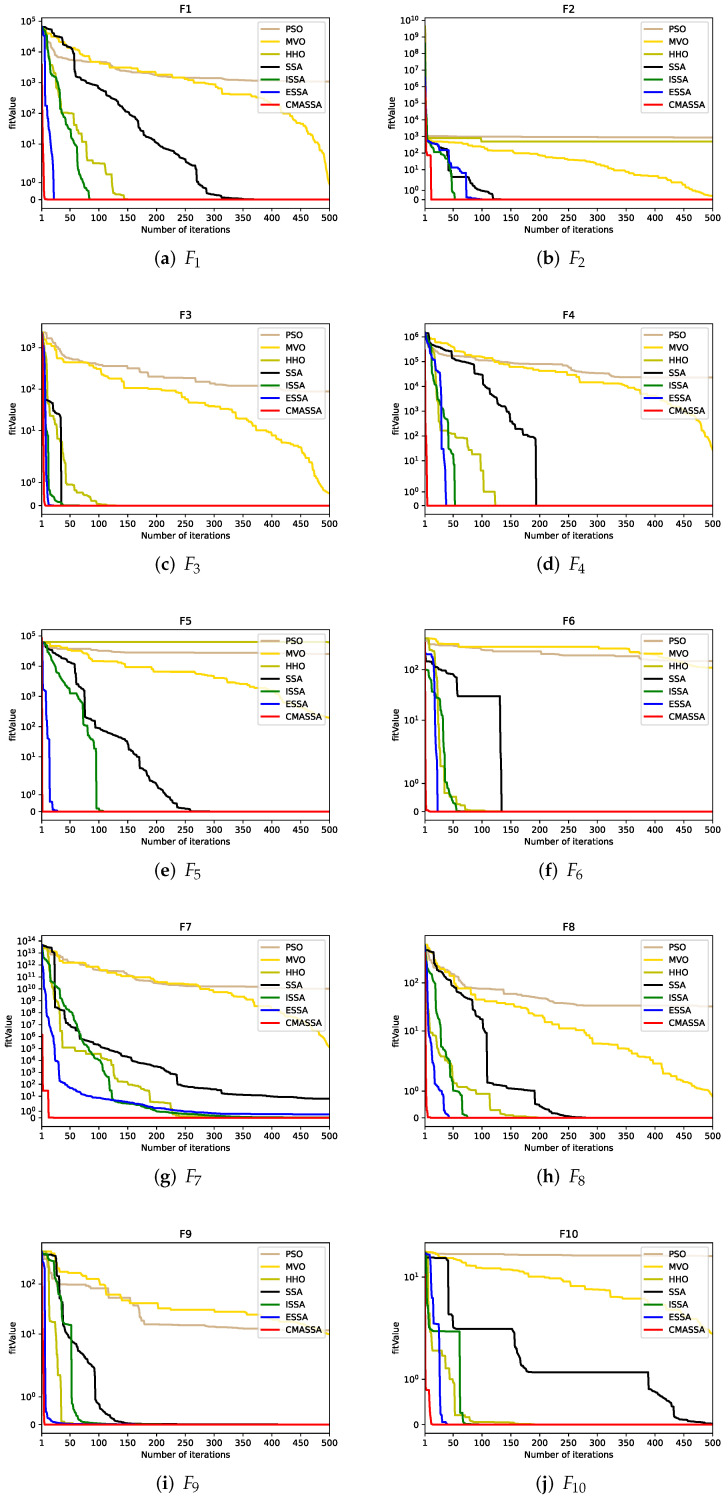
Convergence curves of seven algorithms on ten test functions.

To ensure the accuracy of the comparative experiments, the population number of each algorithm is set to 30, the number of iterations is 500, and the dimension and search space range are set according to the benchmark functions in Table 1. To increase the persuasiveness of the experimental results, each function was tested 30 times, and the standard deviation, average value, optimal value, worst value, and running time were calculated from the optimal value of each function run. The final results are shown in Table 3. For visual comparison, the convergence curves of the seven optimization algorithms on the ten benchmark functions are shown in Figure 7.

It can be seen from the test results in Table 3 that, first of all, in terms of the execution time of the algorithm, although the execution time of CMASSA is slightly slower than that of SSA, it is obviously better than the comparison algorithm. Secondly, on the 10 benchmark functions, CMASSA can reach the theoretical optimal value on $F_{1}$, $F_{2}$, $F_{3}$, $F_{4}$, $F_{5}$, $F_{6}$, and $F_{8}$, and the STD is much smaller than other comparison algorithms. The STD of solving $F_{4}$, $F_{6}$, $F_{8}$, and $F_{10}$ is 0. When solving STD, MEAN and BEST on $F_{7}$ and $F_{9}$, it exceeds other algorithms by at least two orders of magnitude.

Therefore, it is demonstrated that in the scenarios tested, CMASSA has stronger optimization ability, higher stability, and relatively faster execution speed than the comparison algorithms.

Figure 7 clearly shows the changes in the fitness value of each algorithm during the optimization process. It can be clearly seen that the convergence speed of CMASSA is faster than other algorithms. On the high-dimensional test function, as shown in Figure 7f,h,j, SSA has obvious faults, while CMASSA’s faults are not obvious. It shows that after a small number of iterations, CMASSA jumps out of the local optimum, indicating that CMASSA has a stronger ability to jump out of the local optimum.

To sum up, the optimization performance of CMASSA on high-dimensional multi-peak benchmark functions is significantly stronger than other optimization algorithms, and the stability is the strongest. The decreasing speed of the low-dimensional benchmark function is obvious, which proves that CMASSA enhances the local search ability of the standard SSA algorithm and improves the optimization speed.

### 5.3. Data Compression and Reconstruction on Multi-Class Weather Dataset

#### 5.3.1. Initialization of the Optimization Algorithm

This experiment compares CAEN hyperparameter optimization based on CMASSA (CMASSA-CAEN), CAEN hyperparameter optimization based on particle swarm optimization (PSO-CAEN) [29], and CAEN hyperparameter optimization based on a sparrow search algorithm (SSA-CAEN).

The initial parameters of the CMASSA are set as shown in Table 4.

The initial parameters of the PSO are set as shown in Table 5.

#### 5.3.2. CAEN Model Structure

The experiments in this paper compare the performance of the CAEN models output by each optimization algorithm on the Multi-class Weather Dataset (MWD) [30] of Shenzhen University. MWD, which contains 65,000 images in six common categories of sunny, cloudy, rainy, snowy, haze, and thunderstorms. For each model training, 70% are randomly selected as the training set, and the remaining 30% are used as the test set. The experiment relies on the SCM artificial intelligence cloud platform of Dalian University, uses the Python deep learning library Keras framework to build the CAEN model structure.

Considering the training cost of deep learning, this paper does not set too many CAEN layers. The CCN in CAEN consists of two convolution pooling layers, and the CDN consists of two deconvolution upsampling layers. To ensure that the input is reconstructed as much as possible. Therefore, the convolutional layer in CCN is set to be the same as the deconvolutional layer hyperparameters of CDN, and the pooling layer of CCN is set to be the same as the upsampling layer hyperparameters of CDN.

**Figure 8:**
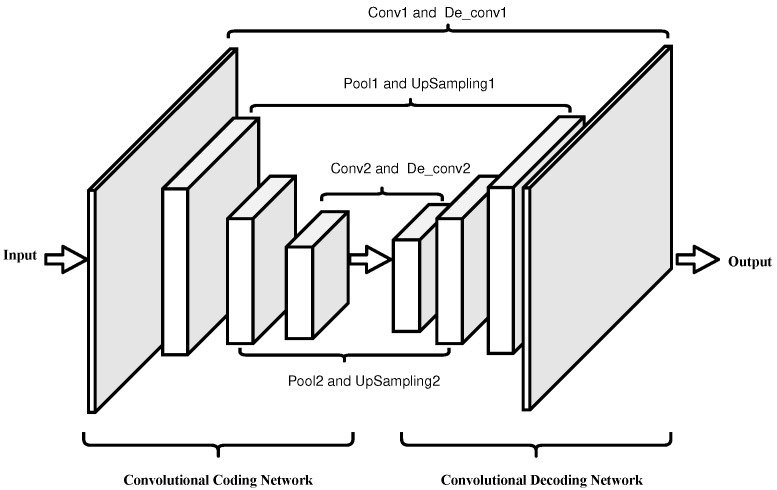
The structure of CAEN.

The structure of CAEN is shown in Figure 8.

As shown in Table 6, in the above CAEN model, since CCN and CDN are inverse processes of each other, it is necessary to set the parameters of the convolutional layer of CCN and the deconvolutional layer of CDN to be the same; likewise, the parameters of the pooling layer of CCN and the upsampling layer of CDN need to be set to be the same. As the pooling kernel size hyperparameter of the pooling layer determines the compression ratio of the data, as shown in Equation (12), the compression ratio of the CAEN model established in this paper is between 1/4 and 1/64.

(12) $$C_{r}=1/\prod_{k}^{s}k$$

where $C_{r}$ represents the compression ratio of CAEN, *s* represents the number of pooling layers of CAEN, and *k* represents the size of the pooling kernel in the current pooling layer.

#### 5.3.3. Time Complexity Analysis of Optimization Algorithm

To verify that the performance benefits of CMASSA are not gained at the expense of time, CMASSA and SSA are compared. According to the literature [31], the time complexity of SSA is:

(13) T=O(D+f(D))

where *D* represents the dimension, f(D) represents the time required to solve the objective function.

Assuming that the population size in the algorithm is *N*, the dimension is Dim, the maximum number of iterations is $T_{m}ax$, the time to randomly initialize the population parameters is $s_{1}$, and the time to find the individual fitness value is t(Dim), explore The number of followers is pNum, the update time of each dimension is $s_{2}$, the number of followers is sNum, the update time of each dimension is $s_{3}$, and the update time of each dimension of the alerter is $s_{4}$.

In CMASSA, Assuming to improve the Circle Map to enhance the diversity of the population, the time required is $u_{1}$, so the time complexity of the initial stage is $T_{1}=O\left(s_{1}+N\left(u_{1}+t\left(Dim\right)+s_{1}Dim\right)\right)$, executing the finder and The time required to update the formula for the number of followers is $u_{3}$. The update time complexity of the discoverer is: $T_{2}=O\left(s_{2}pNumDim+u_{3}T_{max}\right)$, the update time complexity of the follower is: $T_{3}=O\left(s_{3}sNumDim+u_{3}T_{max}\right)$, the update time complexity of the alerter is: $T_{4}=O\left(s_{4}\left(N-pNum-sNum\right)Dim\right)$.

To sum up, the time complexity of CMASSA is: $T=T_{1}+\left(T_{2}+T_{3}+T_{4}\right)T_{max}=O\left(Dim+t\left(Dim\right)\right)$, indicating that the time complexity of CMASSA does not increase.

#### 5.3.4. Experimental Results

The results shown in Table 7, the optimal hyperparameters of CAEN output by each optimization algorithm on MWD. It can be seen from Equation (12) that the optimal CAEN network structure output by CMASSA can achieve a maximum compression ratio of 1/32 when balancing the performance of the model to perform the classification task and the degree of data reconstruction.

According to the two items of X3 and X6 in Table 7, the maximum compression ratio that can be achieved by CAEN of the output of each optimization algorithm is calculated, as shown in Table 8.

Table 9 shows the highest accuracy of each optimization algorithm to optimize the CAEN model on the MWD test set. Compared with other optimization algorithms, the optimization effect of the CMASSA-CAEN algorithm is remarkable.

**Figure 9:**
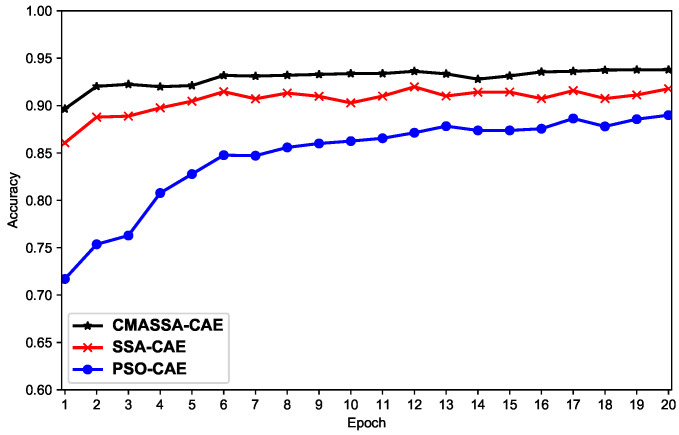
The accuracy of each optimization algorithm on the classification task.

Figure 9 is a graph showing the change trend of the classification accuracy of CAEN optimized by each optimization algorithm under 20 epochs. The network model obtained by the CMASSA-CAEN training algorithm is characterized, and the accuracy rate on the test set is higher than other algorithms, which proves that the performance of the CMASSA-CAEN algorithm is indeed better than other optimization algorithms. It can also be seen that at the beginning of training, the CAEN accuracy rate obtained by the CMASSA is significantly higher than that of other optimization algorithms, which proves that the network model established by CMASSA-CAEN has stronger optimization ability and more stable performance.

In addition to the classification accuracy, the data reconstruction degree of the model is also an important indicator to characterize the pros and cons of the CAEN model. Using Equation (11) to calculate the data reconstruction degree, as shown in Table 10, the data reconstruction degree of the CMASSA-CAEN algorithm is significantly higher than that of other optimization algorithms, reaching 99.41%, indicating that when the data size is compressed to 1/32, the data after recovery is still such that most of the information can be retained.

To sum up, CMASSA-CAEN can still achieve the highest data reconstruction degree and accuracy under the premise that the data compression ratio reaches 1/32. Although the result of PSO-CAEN achieves a compression ratio of 1/32, it has the worst performance in both performance metrics. Prove that CMASSA outperforms other algorithms.

**Figure 10:**
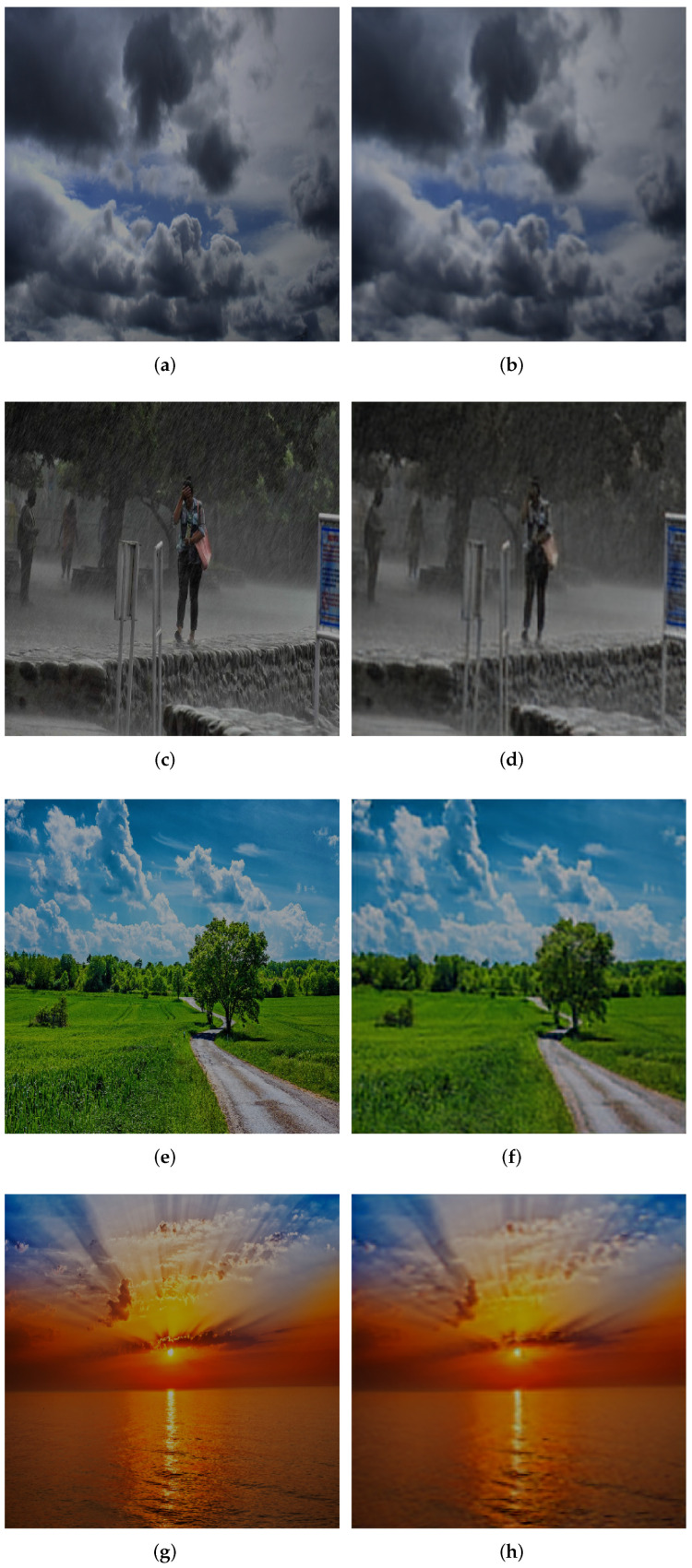
The original image and its compressed and reconstructed image. (**a**) Cloudy Original Image; (**b**) Cloudy Compression Reconstruction Image; (**c**) Rain Original Image; (**d**) Rain Compression Reconstruction Image; (**e**) Shine Original Image; (**f**) Shine Compression Reconstruction Image; (**g**) Sunrise Original Image; (**h**) Sunrise Compression Reconstruction Image.

As shown in Figure 10 below, the data compression of CMASSA-CAEN performed on MWD is compared with the original image.

## 6. Conclusions

This paper proposes a novel chaotic mutation adaptive sparrow search algorithm (CMASSA), which is an excellent improvement on the sparrow search algorithm (SSA). The paper applies CMASSA to data compression. The idea is to build a new fitness function, and use the CMASSA to optimize the CAEN network hyperparameters in the cloud service center. The optimal network model obtained by training is sent to the edge server to compress the edge data. First, aiming at the problem of insufficient population diversity in the later stage of SSA, an improved Circle Map is introduced to enhance the diversity of the initial population; secondly, the sine and cosine mutation operator is introduced into the sparrow alerters equation to enhance the local development ability. To avoid falling into a local optimum early, an adaptive population adjustment strategy is proposed to adjust the ratio of discoverers and followers to balance the global search ability and local optimization ability of the sparrow search algorithm. Finally, the test results on multiple benchmark functions are excellent. Through comparative experiments, it is demonstrated that the data compressed by CMASSA-CAEN, with the compression ratio as high as 1/32, the accuracy of the classification task is significantly stronger than other optimization algorithms of the same type, and when the data is restored and reconstructed, the data reconstruction degree is as high as 99.41%, far exceeding other algorithms. 

## Figures and Tables

**Table 1:** Benchmark functions.

Function Equation	Search Space and Dimension	Best Value
$F_{1}\left(x\right)=\sum_{i=1}^{d}x_{i}^{2}$	[−500, 500, 30]	0
$F_{2}\left(x\right)=\sum_{i=1}^{d}x_{i}^{2}+{\left(\sum_{i=1}^{d}0.5ix_{i}\right)}^{2}+{\left(\sum_{i=1}^{d}0.5ix_{i}\right)}^{4}$	[−5, 10, 30]	0
$F_{3}\left(x\right)=\sum_{i=1}^{d}i\cdot x_{i}^{2}$	[−5.12, 5.12, 30]	0
$F_{4}\left(x\right)=\sum_{i=1}^{d}{\left(\left\lfloor x_{i}+0.5\right\rfloor \right)}^{2}$	[−5.12, 5.12, 30]	0
$F_{5}\left(x\right)=\sum_{i=1}^{d}{\left(\sum_{j=1}^{d}x_{j}\right)}^{2}$	[−100, 100, 30]	0
$F_{6}\left(x\right)=\sum_{i=1}^{d}\left[x_{i}^{2}-10\cos\left(2\pi x_{i}\right)+10\right]$	[−5.12, 5.12, 30]	0
$F_{7}\left(x\right)=\sum_{i=1}^{d-1}\left[100{\left(x_{i+1}-x_{i}^{2}\right)}^{2}+{\left(x_{i}-1\right)}^{2}\right]$	[−600, 600, 30]	0
$F_{8}\left(x\right)=\frac{1}{4000}\sum_{i=1}^{d}x_{i}^{2}-\prod_{i=1}^{d}\cos\left(\frac{x_{i}}{\sqrt{i}}\right)+1$	[−600, 600, 30]	0
$\begin{array}{cc} & F_{9}\left(x\right)=\frac{\pi }{n}\left\{10\sin\left(\pi y_{1}\right)+\sum_{i=1}^{d}{\left(y_{i}-1\right)}^{2}\left[1+10\sin^{2}\left(\pi y_{i+1}\right)\right]+{\left(y_{n}-1\right)}^{2}\right\}\\ & +\sum_{i=1}^{d}u\left(x_{i},10,100,4\right)\\ & \mathrm{where}:\;y_{i}=1+\frac{x_{i}+1}{4},u\left(x_{i},a,k,m\right)=\left\{\begin{array}{c} k{\left(x_{i}-a\right)}^{m}x_{i}>a\\ 0-a<x_{i}<a\\ k{\left(-x_{i}-a\right)}^{m}x_{i}<-a \end{array}\right. \end{array}$	[−50, 50, 30]	0
$F_{10}\left(x\right)=-20\exp\left(-0.2\sqrt{\frac{1}{d}\sum_{i=1}^{d}x_{i}^{2}}\right)-\exp\left(\frac{1}{d}\sum_{i=1}^{d}\cos\left(2\pi x_{i}\right)\right)+20+\exp\left(1\right)$	[−32, 32, 30]	0

**Table 2:** Ablation experiment comparison table.

ID	INDEX	CMASSA	SSA	SSA1	SSA2	SSA3
	STD	$1.0705\times 10^{-85}$	$6.5600\times 10^{-05}$	$5.9768\times 10^{-05}$	$4.5491\times 10^{-70}$	$8.9619\times 10^{-20}$
	MEAN	$2.1479\times 10^{-86}$	$1.5155\times 10^{-05}$	$1.3201\times 10^{-05}$	$8.5759\times 10^{-71}$	$1.6833\times 10^{-20}$
$F_{1}$	BEST	$0.0000\times 10^{+00}$	$1.1051\times 10^{-35}$	$2.1040\times 10^{-17}$	$0.0000\times 10^{+00}$	$0.0000\times 10^{+00}$
	WORST	$5.9615\times 10^{-85}$	$3.5868\times 10^{-04}$	$3.3458\times 10^{-04}$	$2.5353\times 10^{-69}$	$4.9942\times 10^{-19}$
	TIME	$1.2221\times 10^{+00}$	$1.1846\times 10^{+00}$	$1.1841\times 10^{+00}$	$1.2154\times 10^{+00}$	$1.1568\times 10^{+00}$
	STD	$2.0090\times 10^{-61}$	$9.4905\times 10^{-10}$	$1.8694\times 10^{-07}$	$8.5809\times 10^{-48}$	$5.7652\times 10^{-16}$
	MEAN	$3.7340\times 10^{-62}$	$2.5074\times 10^{-10}$	$3.5786\times 10^{-08}$	$1.5934\times 10^{-48}$	$1.0751\times 10^{-16}$
$F_{2}$	BEST	$0.0000\times 10^{+00}$	$5.3019\times 10^{-149}$	$1.2225\times 10^{-26}$	$4.3399\times 10^{-110}$	$8.7699\times 10^{-119}$
	WORST	$1.1192\times 10^{-60}$	$4.3303\times 10^{-09}$	$1.0421\times 10^{-06}$	$4.7803\times 10^{-47}$	$3.2122\times 10^{-15}$
	TIME	$2.1157\times 10^{+00}$	$2.0658\times 10^{+00}$	$2.0742\times 10^{+00}$	$2.0835\times 10^{+00}$	$2.0402\times 10^{+00}$
	STD	$2.7895\times 10^{-82}$	$6.5093\times 10^{-11}$	$9.3050\times 10^{-13}$	$1.8913\times 10^{-69}$	$1.0286\times 10^{-23}$
	MEAN	$5.1824\times 10^{-83}$	$2.9111\times 10^{-11}$	$4.6638\times 10^{-13}$	$3.6717\times 10^{-70}$	$5.3750\times 10^{-24}$
$F_{3}$	BEST	$0.0000\times 10^{+00}$	$3.9000\times 10^{-28}$	$7.9414\times 10^{-20}$	$3.9929\times 10^{-221}$	$6.2726\times 10^{-90}$
	WORST	$1.5540\times 10^{-81}$	$1.7466\times 10^{-10}$	$2.3274\times 10^{-12}$	$1.0546\times 10^{-68}$	$2.5935\times 10^{-23}$
	TIME	$1.6982\times 10^{+00}$	$1.6567\times 10^{+00}$	$1.6550\times 10^{+00}$	$1.6880\times 10^{+00}$	$1.6347\times 10^{+00}$
	STD	$0.0000\times 10^{+00}$	$0.0000\times 10^{+00}$	$0.0000\times 10^{+00}$	$0.0000\times 10^{+00}$	$0.0000\times 10^{+00}$
	MEAN	$0.0000\times 10^{+00}$	$0.0000\times 10^{+00}$	$0.0000\times 10^{+00}$	$0.0000\times 10^{+00}$	$0.0000\times 10^{+00}$
$F_{4}$	BEST	$0.0000\times 10^{+00}$	$0.0000\times 10^{+00}$	$0.0000\times 10^{+00}$	$0.0000\times 10^{+00}$	$0.0000\times 10^{+00}$
	WORST	$0.0000\times 10^{+00}$	$0.0000\times 10^{+00}$	$0.0000\times 10^{+00}$	$0.0000\times 10^{+00}$	$0.0000\times 10^{+00}$
	TIME	$1.2673\times 10^{+00}$	$1.2138\times 10^{+00}$	$1.2133\times 10^{+00}$	$1.2650\times 10^{+00}$	$1.2006\times 10^{+00}$
	STD	$1.5747\times 10^{-69}$	$1.0348\times 10^{-07}$	$1.6461\times 10^{-07}$	$6.8243\times 10^{-63}$	$2.6072\times 10^{-14}$
	MEAN	$2.9242\times 10^{-70}$	$5.3185\times 10^{-08}$	$5.2568\times 10^{-08}$	$1.4476\times 10^{-63}$	$1.0942\times 10^{-14}$
$F_{5}$	BEST	$0.0000\times 10^{+00}$	$3.4167\times 10^{-22}$	$1.5463\times 10^{-88}$	$0.0000\times 10^{+00}$	$1.1767\times 10^{-81}$
	WORST	$8.7725\times 10^{-69}$	$2.8275\times 10^{-07}$	$6.6255\times 10^{-07}$	$3.7801\times 10^{-62}$	$8.7516\times 10^{-14}$
	TIME	$4.2571\times 10^{+00}$	$4.2185\times 10^{+00}$	$4.2133\times 10^{+00}$	$4.2526\times 10^{+00}$	$4.1772\times 10^{+00}$
	STD	$0.0000\times 10^{+00}$	$3.4843\times 10^{-09}$	$1.8214\times 10^{-05}$	$0.0000\times 10^{+00}$	$6.0585\times 10^{-15}$
	MEAN	$0.0000\times 10^{+00}$	$8.0047\times 10^{-10}$	$3.3824\times 10^{-06}$	$0.0000\times 10^{+00}$	$1.1250\times 10^{-15}$
$F_{6}$	BEST	$0.0000\times 10^{+00}$	$0.0000\times 10^{+00}$	$0.0000\times 10^{+00}$	$0.0000\times 10^{+00}$	$0.0000\times 10^{+00}$
	WORST	$0.0000\times 10^{+00}$	$1.8935\times 10^{-08}$	$1.0147\times 10^{-04}$	$0.0000\times 10^{+00}$	$3.3751\times 10^{-14}$
	TIME	$3.0034\times 10^{+00}$	$2.9302\times 10^{+00}$	$2.9384\times 10^{+00}$	$2.9666\times 10^{+00}$	$2.9140\times 10^{+00}$
	STD	$1.6476\times 10^{-03}$	$2.8969\times 10^{-02}$	$3.8748\times 10^{+00}$	$7.6422\times 10^{-01}$	$3.9448\times 10^{-01}$
	MEAN	$7.8224\times 10^{-03}$	$2.2073\times 10^{-02}$	$5.2807\times 10^{+00}$	$5.1171\times 10^{-01}$	$3.8762\times 10^{-01}$
$F_{7}$	BEST	$8.1175\times 10^{-12}$	$1.1745\times 10^{-06}$	$8.0121\times 10^{-02}$	$5.9266\times 10^{-12}$	$2.3565\times 10^{-07}$
	WORST	$7.0049\times 10^{+00}$	$7.4449\times 10^{+00}$	$9.2716\times 10^{+00}$	$2.7046\times 10^{+00}$	$1.0360\times 10^{+00}$
	TIME	$2.5757\times 10^{+00}$	$2.5306\times 10^{+00}$	$2.5235\times 10^{+00}$	$2.5548\times 10^{+00}$	$2.5001\times 10^{+00}$
	STD	$0.0000\times 10^{+00}$	$1.0096\times 10^{-06}$	$1.2464\times 10^{-04}$	$0.0000\times 10^{+00}$	$0.0000\times 10^{+00}$
	MEAN	$0.0000\times 10^{+00}$	$5.4374\times 10^{-06}$	$6.7123\times 10^{-04}$	$0.0000\times 10^{+00}$	$0.0000\times 10^{+00}$
$F_{8}$	BEST	$0.0000\times 10^{+00}$	$3.5430\times 10^{-10}$	$0.0000\times 10^{+00}$	$0.0000\times 10^{+00}$	$0.0000\times 10^{+00}$
	WORST	$0.0000\times 10^{+00}$	$5.6249\times 10^{-06}$	$6.9438\times 10^{-04}$	$0.0000\times 10^{+00}$	$0.0000\times 10^{+00}$
	TIME	$3.7824\times 10^{+00}$	$3.7233\times 10^{+00}$	$3.7119\times 10^{+00}$	$3.7636\times 10^{+00}$	$3.7007\times 10^{+00}$
	STD	$9.5208\times 10^{-07}$	$2.3760\times 10^{-03}$	$1.9280\times 10^{+01}$	$6.7245\times 10^{-04}$	$1.1718\times 10^{-03}$
	MEAN	$1.5477\times 10^{-07}$	$4.0611\times 10^{-03}$	$7.2792\times 10^{+00}$	$1.0828\times 10^{-03}$	$1.5485\times 10^{-03}$
$F_{9}$	BEST	$1.1659\times 10^{-13}$	$1.5555\times 10^{-06}$	$4.3073\times 10^{-11}$	$1.1659\times 10^{-13}$	$1.0080\times 10^{-10}$
	WORST	$2.9828\times 10^{-04}$	$1.1858\times 10^{-02}$	$6.4671\times 10^{+01}$	$2.3158\times 10^{-03}$	$3.5032\times 10^{-03}$
	TIME	$2.6967\times 10^{+00}$	$2.6250\times 10^{+00}$	$2.6291\times 10^{+00}$	$2.6813\times 10^{+00}$	$2.6050\times 10^{+00}$
	STD	$0.0000\times 10^{+00}$	$9.2665\times 10^{-04}$	$4.9267\times 10^{-01}$	$0.0000\times 10^{+00}$	$7.9115\times 10^{-09}$
	MEAN	$4.4409\times 10^{-16}$	$1.7345\times 10^{-04}$	$3.7489\times 10^{-01}$	$4.4409\times 10^{-16}$	$5.0084\times 10^{-09}$
$F_{10}$	BEST	$4.4409\times 10^{-16}$	$3.9968\times 10^{-15}$	$4.4409\times 10^{-16}$	$4.4409\times 10^{-16}$	$7.5495\times 10^{-15}$
	WORST	$4.4409\times 10^{-16}$	$5.1636\times 10^{-03}$	$1.0320\times 10^{+00}$	$4.4409\times 10^{-16}$	$2.0336\times 10^{-08}$
	TIME	$1.7532\times 10^{+00}$	$1.7479\times 10^{+00}$	$1.7102\times 10^{+00}$	$1.7288\times 10^{+00}$	$1.6958\times 10^{+00}$

**Table 3:** Comparison of results of optimization algorithm test benchmark functions.

ID	INDEX	CMASSA	SSA	ISSA	ESSA	HHO	MVO	PSO
	STD	$1.0705\times 10^{-85}$	$6.5600\times 10^{-05}$	$5.0970\times 10^{-18}$	$5.4102\times 10^{-14}$	$2.5505\times 10^{-40}$	$3.4103\times 10^{-01}$	$5.4014\times 10^{+03}$
	MEAN	$2.1479\times 10^{-86}$	$1.5155\times 10^{-05}$	$1.5132\times 10^{-18}$	$1.4515\times 10^{-14}$	$5.3741\times 10^{-41}$	$1.1173\times 10^{+00}$	$5.0247\times 10^{+03}$
$F_{1}$	BEST	$0.0000\times 10^{+00}$	$1.1051\times 10^{-35}$	$1.0385\times 10^{-90}$	$1.8330\times 10^{-94}$	$8.8868\times 10^{-67}$	$6.6420\times 10^{-01}$	$6.3746\times 10^{+01}$
	WORST	$5.9615\times 10^{-85}$	$3.5868\times 10^{-04}$	$2.0545\times 10^{-17}$	$2.8776\times 10^{-13}$	$1.4138\times 10^{-39}$	$1.9594\times 10^{+00}$	$2.1421\times 10^{+04}$
	TIME	$1.2221\times 10^{+00}$	$1.1846\times 10^{+00}$	$1.3623\times 10^{+00}$	$1.3747\times 10^{+00}$	$6.9779\times 10^{+00}$	$7.2620\times 10^{+00}$	$1.0651\times 10^{+01}$
	STD	$2.0090\times 10^{-61}$	$9.4905\times 10^{-10}$	$9.8620\times 10^{-15}$	$3.6959\times 10^{-06}$	$1.0269\times 10^{+02}$	$2.1911\times 10^{-01}$	$1.8001\times 10^{+02}$
	MEAN	$3.7340\times 10^{-62}$	$2.5074\times 10^{-10}$	$3.2257\times 10^{-15}$	$6.8805\times 10^{-07}$	$4.7583\times 10^{+02}$	$4.6434\times 10^{-01}$	$4.3397\times 10^{+02}$
$F_{2}$	BEST	$0.0000\times 10^{+00}$	$5.3019\times 10^{-149}$	$4.5545\times 10^{-30}$	$1.0942\times 10^{-28}$	$3.0137\times 10^{+02}$	$1.8423\times 10^{-01}$	$1.0650\times 10^{+02}$
	WORST	$1.1192\times 10^{-60}$	$4.3303\times 10^{-09}$	$4.7048\times 10^{-14}$	$2.0591\times 10^{-05}$	$6.2415\times 10^{+02}$	$1.3057\times 10^{+00}$	$7.5349\times 10^{+02}$
	TIME	$2.1157\times 10^{+00}$	$2.0658\times 10^{+00}$	$2.5193\times 10^{+00}$	$2.4678\times 10^{+00}$	$1.1002\times 10^{+01}$	$8.1206\times 10^{+00}$	$1.1883\times 10^{+01}$
	STD	$2.7895\times 10^{-82}$	$6.5093\times 10^{-11}$	$4.4623\times 10^{-18}$	$1.9940\times 10^{-11}$	$2.3194\times 10^{-44}$	$2.3126\times 10^{-01}$	$2.0029\times 10^{+02}$
	MEAN	$5.1824\times 10^{-83}$	$2.9111\times 10^{-11}$	$1.2281\times 10^{-18}$	$3.8592\times 10^{-12}$	$5.5736\times 10^{-45}$	$2.3559\times 10^{-01}$	$1.7617\times 10^{+02}$
$F_{3}$	BEST	$0.0000\times 10^{+00}$	$3.9000\times 10^{-28}$	$2.0856\times 10^{-25}$	$3.9675\times 10^{-26}$	$3.0741\times 10^{-62}$	$6.3075\times 10^{-02}$	$9.9219\times 10^{+00}$
	WORST	$1.5540\times 10^{-81}$	$1.7466\times 10^{-10}$	$2.3858\times 10^{-17}$	$1.1117\times 10^{-10}$	$1.2302\times 10^{-43}$	$1.0659\times 10^{+00}$	$1.0805\times 10^{+03}$
	TIME	$1.6982\times 10^{+00}$	$1.6567\times 10^{+00}$	$2.0883\times 10^{+00}$	$2.0787\times 10^{+00}$	$7.6067\times 10^{+00}$	$8.1141\times 10^{+00}$	$1.1672\times 10^{+01}$
	STD	$0.0000\times 10^{+00}$	$0.0000\times 10^{+00}$	$0.0000\times 10^{+00}$	$0.0000\times 10^{+00}$	$0.0000\times 10^{+00}$	$1.9400\times 10^{+01}$	$9.4794\times 10^{+04}$
	MEAN	$0.0000\times 10^{+00}$	$0.0000\times 10^{+00}$	$0.0000\times 10^{+00}$	$0.0000\times 10^{+00}$	$0.0000\times 10^{+00}$	$4.7700\times 10^{+01}$	$7.0315\times 10^{+04}$
$F_{4}$	BEST	$0.0000\times 10^{+00}$	$0.0000\times 10^{+00}$	$0.0000\times 10^{+00}$	$0.0000\times 10^{+00}$	$0.0000\times 10^{+00}$	$1.9000\times 10^{+01}$	$5.0350\times 10^{+03}$
	WORST	$0.0000\times 10^{+00}$	$0.0000\times 10^{+00}$	$0.0000\times 10^{+00}$	$0.0000\times 10^{+00}$	$0.0000\times 10^{+00}$	$1.1400\times 10^{+02}$	$4.4366\times 10^{+05}$
	TIME	$1.2673\times 10^{+00}$	$1.2138\times 10^{+00}$	$1.5161\times 10^{+00}$	$1.4879\times 10^{+00}$	$1.0426\times 10^{+01}$	$7.5642\times 10^{+00}$	$1.1148\times 10^{+01}$
	STD	$1.5747\times 10^{-69}$	$1.0348\times 10^{-07}$	$1.1735\times 10^{-14}$	$2.6894\times 10^{-08}$	$3.1017\times 10^{+04}$	$1.1204\times 10^{+02}$	$1.1643\times 10^{+04}$
	MEAN	$2.9242\times 10^{-70}$	$5.3185\times 10^{-08}$	$2.3675\times 10^{-15}$	$5.5321\times 10^{-09}$	$1.1793\times 10^{+05}$	$2.3648\times 10^{+02}$	$2.6290\times 10^{+04}$
$F_{5}$	BEST	$0.0000\times 10^{+00}$	$3.4167\times 10^{-22}$	$8.8437\times 10^{-90}$	$1.2052\times 10^{-88}$	$5.3578\times 10^{+04}$	$8.2378\times 10^{+01}$	$7.0251\times 10^{+03}$
	WORST	$8.7725\times 10^{-69}$	$2.8275\times 10^{-07}$	$6.5477\times 10^{-14}$	$1.4952\times 10^{-07}$	$1.9696\times 10^{+05}$	$6.0401\times 10^{+02}$	$4.9277\times 10^{+04}$
	TIME	$4.2571\times 10^{+00}$	$4.2185\times 10^{+00}$	$5.3255\times 10^{+00}$	$5.2709\times 10^{+00}$	$1.3209\times 10^{+01}$	$1.0619\times 10^{+01}$	$1.4266\times 10^{+01}$
	STD	$0.0000\times 10^{+00}$	$3.4843\times 10^{-09}$	$3.1887\times 10^{-16}$	$2.3918\times 10^{-10}$	$6.3773\times 10^{-16}$	$3.1278\times 10^{+01}$	$3.3572\times 10^{+01}$
	MEAN	$0.0000\times 10^{+00}$	$8.0047\times 10^{-10}$	$5.9212\times 10^{-17}$	$9.5296\times 10^{-11}$	$1.1842\times 10^{-16}$	$1.3775\times 10^{+02}$	$1.5238\times 10^{+02}$
$F_{6}$	BEST	$0.0000\times 10^{+00}$	$0.0000\times 10^{+00}$	$0.0000\times 10^{+00}$	$0.0000\times 10^{+00}$	$0.0000\times 10^{+00}$	$8.4781\times 10^{+01}$	$8.5800\times 10^{+01}$
	WORST	$0.0000\times 10^{+00}$	$1.8935\times 10^{-08}$	$1.7764\times 10^{-15}$	$8.0990\times 10^{-10}$	$3.5527\times 10^{-15}$	$2.1294\times 10^{+02}$	$2.4324\times 10^{+02}$
	TIME	$3.0034\times 10^{+00}$	$2.9302\times 10^{+00}$	$3.4896\times 10^{+00}$	$3.4849\times 10^{+00}$	$1.1015\times 10^{+01}$	$8.9346\times 10^{+00}$	$1.2328\times 10^{+01}$
	STD	$1.6476\times 10^{-03}$	$2.8969\times 10^{-02}$	$2.3439\times 10^{-02}$	$9.7064\times 10^{-02}$	$4.9368\times 10^{+00}$	$9.8644\times 10^{+06}$	$1.3224\times 10^{+11}$
	MEAN	$7.8224\times 10^{-03}$	$2.2073\times 10^{-02}$	$1.3104\times 10^{-02}$	$4.6946\times 10^{-02}$	$9.2151\times 10^{-01}$	$6.9597\times 10^{+06}$	$6.0178\times 10^{+10}$
$F_{7}$	BEST	$8.1175\times 10^{-12}$	$1.1745\times 10^{-06}$	$2.1312\times 10^{-09}$	$1.3492\times 10^{-07}$	$6.3521\times 10^{-04}$	$3.9736\times 10^{+04}$	$5.0400\times 10^{+06}$
	WORST	$7.0049\times 10^{+00}$	$7.4449\times 10^{+00}$	$9.4335\times 10^{-02}$	$4.6223\times 10^{-01}$	$2.7507\times 10^{+01}$	$3.1250\times 10^{+07}$	$6.6952\times 10^{+11}$
	TIME	$2.5757\times 10^{+00}$	$2.5306\times 10^{+00}$	$2.9729\times 10^{+00}$	$2.9691\times 10^{+00}$	$1.0661\times 10^{+01}$	$8.4577\times 10^{+00}$	$1.2021\times 10^{+01}$
	STD	$0.0000\times 10^{+00}$	$1.0096\times 10^{-06}$	$1.2324\times 10^{-16}$	$6.1255\times 10^{-14}$	$1.6126\times 10^{-01}$	$4.9967\times 10^{-02}$	$3.5283\times 10^{+01}$
	MEAN	$0.0000\times 10^{+00}$	$5.4374\times 10^{-06}$	$4.0708\times 10^{-17}$	$1.8404\times 10^{-14}$	$4.2527\times 10^{-02}$	$8.7066\times 10^{-01}$	$2.6632\times 10^{+01}$
$F_{8}$	BEST	$0.0000\times 10^{+00}$	$3.5430\times 10^{-10}$	$0.0000\times 10^{+00}$	$0.0000\times 10^{+00}$	$0.0000\times 10^{+00}$	$7.7220\times 10^{-01}$	$1.7997\times 10^{+00}$
	WORST	$0.0000\times 10^{+00}$	$5.6249\times 10^{-06}$	$4.4409\times 10^{-16}$	$3.0975\times 10^{-13}$	$7.3933\times 10^{-01}$	$9.6112\times 10^{-01}$	$1.2635\times 10^{+02}$
	TIME	$3.7824\times 10^{+00}$	$3.7233\times 10^{+00}$	$4.3969\times 10^{+00}$	$4.3939\times 10^{+00}$	$1.1562\times 10^{+01}$	$9.6292\times 10^{+00}$	$1.3236\times 10^{+01}$
	STD	$9.5208\times 10^{-07}$	$2.3760\times 10^{-03}$	$3.8391\times 10^{-04}$	$6.1844\times 10^{-04}$	$6.2743\times 10^{-06}$	$4.5672\times 10^{+01}$	$3.5721\times 10^{+01}$
	MEAN	$1.5477\times 10^{-07}$	$4.0611\times 10^{-03}$	$5.4870\times 10^{-04}$	$8.8340\times 10^{-04}$	$9.1995\times 10^{-06}$	$7.4421\times 10^{+01}$	$4.9296\times 10^{+01}$
$F_{9}$	BEST	$1.1659\times 10^{-13}$	$1.5555\times 10^{-06}$	$4.2957\times 10^{-08}$	$1.8224\times 10^{-06}$	$4.8412\times 10^{-07}$	$8.5071\times 10^{+00}$	$1.5856\times 10^{+00}$
	WORST	$2.9828\times 10^{-04}$	$1.1858\times 10^{-02}$	$1.5917\times 10^{-03}$	$2.1675\times 10^{-03}$	$2.5875\times 10^{-05}$	$1.8366\times 10^{+02}$	$1.4479\times 10^{+02}$
	TIME	$2.6967\times 10^{+00}$	$2.6250\times 10^{+00}$	$3.1554\times 10^{+00}$	$3.1611\times 10^{+00}$	$1.1256\times 10^{+01}$	$8.9603\times 10^{+00}$	$1.2629\times 10^{+01}$
	STD	$0.0000\times 10^{+00}$	$9.2665\times 10^{-04}$	$8.4020\times 10^{-08}$	$3.0140\times 10^{-07}$	$2.6718\times 10^{-15}$	$6.5020\times 10^{-01}$	$5.1978\times 10^{+00}$
	MEAN	$4.4409\times 10^{-16}$	$1.7345\times 10^{-04}$	$3.2309\times 10^{-08}$	$1.2430\times 10^{-07}$	$3.8784\times 10^{-15}$	$2.0168\times 10^{+00}$	$1.5961\times 10^{+01}$
$F_{10}$	BEST	$4.4409\times 10^{-16}$	$3.9968\times 10^{-15}$	$4.3077\times 10^{-14}$	$7.5495\times 10^{-15}$	$4.4409\times 10^{-16}$	$4.9196\times 10^{-01}$	$3.5382\times 10^{+00}$
	WORST	$4.4409\times 10^{-16}$	$5.1636\times 10^{-03}$	$4.1298\times 10^{-07}$	$1.4627\times 10^{-06}$	$7.5495\times 10^{-15}$	$3.7274\times 10^{+00}$	$1.9967\times 10^{+01}$
	TIME	$1.8432\times 10^{+00}$	$1.7479\times 10^{+00}$	$2.0979\times 10^{+00}$	$2.0850\times 10^{+00}$	$9.9648\times 10^{+00}$	$8.0676\times 10^{+00}$	$1.1584\times 10^{+01}$

**Table 4:** CMASSA initial parameters.

Parameter	Value
Population size	100
Iterations	30
Proportion of alerters	20%

**Table 5:** PSO initial parameters.

Parameter	Value
Population size	100
Iterations	30
$c_{1}$ = $c_{2}$	2
Inertia weight *w*	0.7

**Table 6:** The optimization range of some relevant parameters of the CAEN.

Index	Hyperparameters	Search Space
X1	The number of convolution kernels in Conv1 and De_conv1	[1, 128]
X2	The size of the convolution kernel in Conv1 and De_conv1	[2, 4]
X3	Pooling kernel size in Pool1 and UpSampling1	2, 4, 8
X4	The number of convolution kernels in Conv2 and De_conv2	[1, 64]
X5	Convolution kernel size in Conv2 and De_conv2	[2, 4]
X6	Pooling kernel size in Pool2 and UpSampling2	2, 4, 8
X7	Activation function in Conv1 and De_conv1	ReLU, ELU, tanh
X8	Activation function in Conv2 and De_conv2	ReLU, ELU, tanh
X9	proportion of deactivated neurons	[0.1, 0.5]
X10	Batch Size	[16, 128]
X11	Optimizer	Adam, AdaGrad, SGD

**Table 7:** For each optimization algorithm on MWD, the obtained CAEN optimal hyperparameters.

Index	CMASSA Result	SSA Result	PSO Result
X1	124	98	106
X2	2	2	2
X3	4	4	4
X4	64	54	40
X5	2	4	2
X6	8	4	8
X7	ReLU	ReLU	ReLU
X8	ReLU	ReLU	ReLU
X9	0.1	0.1	0.3
X10	32	44	85
X11	Adam	Adam	SGD

**Table 8:** The maximum compression ratio of the optimization algorithm on the MWD.

Index	the Maximum Compression Ratio
CMASSA-CAEN	1/32
SSA-CAEN	1/16
PSO-CAEN	1/32

**Table 9:** The highest classification accuracy of the optimization algorithm on the MWD.

Algorithm	Highest Accuracy	Mean Accuracy
CMASSA-CAEN	0.9379	0.9295
SSA-CAEN	0.9198	0.9057
PSO-CAEN	0.8863	0.8460

**Table 10:** The data reconstruction degree of the optimization algorithm on the MWD.

Algorithm	Data Reconstruction Degree
CMASSA-CAEN	99.41%
SSA-CAEN	97.28%
PSO-CAEN	93.33%

## Data Availability

The processed data required to reproduce these findings cannot be shared as the data also forms part of an ongoing study.
